# Printed and Flexible Microheaters Based on Carbon Nanotubes

**DOI:** 10.3390/nano10091879

**Published:** 2020-09-19

**Authors:** Aniello Falco, Francisco J. Romero, Florin C. Loghin, Alina Lyuleeva, Markus Becherer, Paolo Lugli, Diego P. Morales, Noel Rodriguez, Jose F. Salmerón, Almudena Rivadeneyra

**Affiliations:** 1 Faculty of Science and Technology, Free University of Bolzano, 39100 Bolzano-Bozen, Italy; aniello.falco@unibz.it (A.F.); paolo.lugli@unibz.it (P.L.); 2 Department of Electronics and Computer Technology, University of Granada, 18071 Granada, Spain; franromero@ugr.es (F.J.R.); diegopm@ugr.es (D.P.M.); noel@ugr.es (N.R.); 3 Institute for Nanoelectronics, Technical University of Munich, 80333 Munich, Germany; florin.loghin@tum.de (F.C.L.); markus.becherer@tum.de (M.B.); jf.salmeron@tum.de (J.F.S.); 4 Department of Electronical and Computer Engineering, Rice University, Houston, TX 77005, USA; alina.lyuleeva@rice.edu

**Keywords:** flexible substrate, heater, inkjet printing, printed electronics, silver nanoparticles, SWCNT

## Abstract

This work demonstrates a cost-effective manufacturing method of flexible and fully printed microheaters, using carbon nanotubes (CNTs) as the heating element. Two different structures with different number of CNT layers have been characterized in detail. The benchmarking has been carried out in terms of maximum operating temperature, as well as nominal resistance and input power for different applied voltages. Their performances have been compared with previous reports for similar devices, fabricated with other technologies. The results have shown that the heaters presented can achieve high temperatures in a small area at lower voltages and lower input power. In particular, the fully printed heaters fabricated on a flexible substrate covering an area of 3.2 mm^2^ and operating at 9.5 V exhibit a maximum temperature point above 70 °C with a power consumption below 200 mW. Therefore, we have demonstrated that this technology paves the way for a cost-effective large-scale fabrication of flexible microheaters aimed to be integrated in flexible sensors.

## 1. Introduction

In many different situations, it is necessary to heat up a certain element or area to achieve the expected performance or a correct response. For example, the vast majority of biosensors require a specific temperature to operate [1]: bacteria indicators need a constant temperature in order to allow the bacteria to grow [2], or many gas sensors work at high temperatures to achieve a high selectivity and sensitivity [3,4]. For this reason, it is desirable to design small and low-cost heating elements. These elements should then be easily integrated into the system as close as possible to the area, where the temperature control is required.

In this direction, the use of printed electronics to manufacture such a device is attracting much attention. These techniques provide interesting features—such as flexibility, cost-effective manufacturing, or even biocompatibility [5,6]—which allow their integration in virtually any substrate. Some groups have already developed heaters using this technology. There are examples in the literature where heaters have been manufactured by inkjet printing of silver nanoparticles on polymer films [7,8,9]. However, they are limited by higher operation temperatures because of non-stability caused by electromigration. Additionally, their lifetime is limited by oxidation of the device [10]. Some authors have considered the use of other much more stable metals, such as gold (Au), to enhance the stability of the sensors at variant operating conditions [11]. Recently, Khan et al. have reported aerosol jetted heaters, based on Au-nanoparticles on a polyimide substrate with an area of 0.25 mm^2^. Their micro-hotplates can operate at temperatures up to 250 °C with a power consumption of 39 mW [12]. In other works, the use of silver (AgNW) and copper (CuNWs) nanowires has also been demonstrated for the manufacture of transparent heaters by spray deposition techniques [13,14].

In this work, we explore the use of carbon nanotubes (CNTs) as the heating element for the development of printed and flexible microheaters. CNTs have been already employed for temperature sensing [15,16], as well as gas sensing purposes [17,18]. Recently, we have demonstrated the possibility of recovering a CNT-based gas sensor without external heat source. Instead, DC voltage among its terminals was applied [19], where the CNT layer itself serves as the supplier of the heat energy. In this regard, we have analyzed the features of CNTs as a heating element for their integration in other systems where self-heating is not feasible, but temperature control is needed. CNTs have already been used as heating elements following different fabrication procedures, although most of them are not capable of achieving a direct patterning on the substrate. Examples of them are the CNT-based heaters presented by Ilanchezhiyan et al. [20], which follows a dip coating method, or the spin-coating process reported by Zhout et al. [21]. Other printing techniques, such as screen-printing, are also suitable for the fabrication of CNT heaters, as reported by Sadi et al. [22]. However, inkjet printing presents unique advantages over these previous approaches since, in addition to be suitable for the fabrication of large area flexible heaters and roll-to-roll processes, it also allows the pattering of the substrates without the use of neither screens nor masks, all this with a better resolution when compared with their counterparts [23].

This paper is structured as follows. Section 2 describes the fabrication of the designed heaters. Section 3 presents a detailed characterization in terms of temperature and power for different manufactured devices. Finally, the main conclusions are remarked.

## 2. Materials and Methods

### 2.1. Materials and Fabrication Process

**Figure 1:**
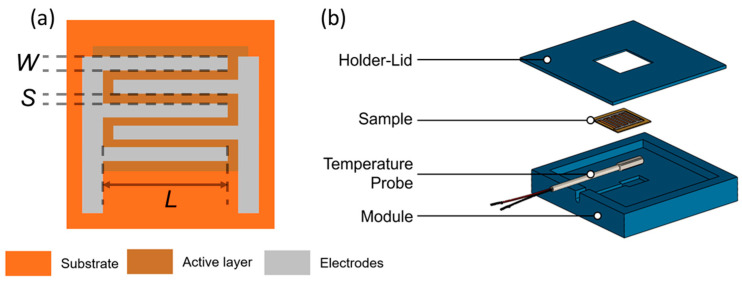
(**a**) Heater schematic. W: fingers’ width, S: separation between both fingers and electrodes, L: length of the fingers. (**b**) Schematic of the measurement setup.

The heaters were fabricated on polyethylene terephthalate (PET) films (Novele Printing Media from Novacentrix, Austin, TX, USA) with 125 μm of thickness. DMP-2831™ Dimatix printer (Fujifilm Dimatix Inc, Santa Clara, USA) was used for the definition of both electrodes and active layer. The substrate temperature was fixed at 60 °C during printing. For the electrodes, a drop space of 40 μm was set in the printer for 80 μm landed diameter drops, whereas the CNT layers were printed at a drop space of 20 μm. The interdigitated electrodes (IDEs) consisted of 1 mm length (L) and 80 μm of both spacing among consecutive fingers (S) and width (W). The total number of electrodes was 20. A simple schematic is depicted in Figure 1a.

First, the electrodes were fabricated by one printing layer of silver ink (DGP 40LT-15C from ANP, Korea) with a solid content of 35% of nanoparticles dispersed in TGME (triethylene glycol monoethyl ether). After drying at 60 °C for 10 min, the patterns were sintered with intense pulsed light (IPL) of a Sinteron 2010 (Xenon, US), fixing one pulse of 2500 μs at 2.50 kV to achieve the lowest resistance and better printing quality [24].

Then, the active layer was inkjet printed on top of the electrodes. For the dispersion of the single-walled CNT (SWCNTs) in aqueous solution, SDS (sodium dodecyl sulphate) is dissolved in deionized water in a weight ratio of 0.5 wt % The SDS solution is stirred for 1 h. The SDS aids with the dispersion of the CNTs as, during the following sonication, the SDS will bind to the CNTs and prevent these from re-bundling. When the solutions is uniform, 0.05 wt % of SWNTs (Hanwha Nanotech) is added and the SDS solution is sonicated for 30 min, respectively, by means of a horn sonicator at 100 W (Branson Sonifier S-450D) to obtain a uniform dispersion of the carbon nanotubes. The CNTs pre sonication have a length range from 5–20 µm with a diameter range from 1.0–1.2 nm. Solutions are finally centrifuged at 15,000 rpm for 90 min and the top 80 % is decanted and used as functional CNT ink [25]. After the deposition of the CNT layer, the sample was dried for 10 min at 60 °C, followed by a bath in deionized (DI) water for 15 min at room temperature to remove the dispersant. Subsequently, it was dried again. Electrical connections were made via gluing wires to the electrodes with silver paint.

### 2.2. Characterization

For the analysis of the heating properties of the fabricated CNT-based devices, a 3D printed module was built. The complete module (Figure 1b) consisted of the device under test (DUT) and a temperature sensor (Pt-100) for the in-situ temperature monitoring. DUT was placed on top of the temperature sensor, which was located in a groove in the carrier part. The sensor was fixed on the carrier part by a lid, which has an opening in the middle to allow the contacting of the sensor. The heaters were driven by a source meter unit (Keithley 2602A Dual-channel SMU) and the resistance was measured with a multimeter (Keithley 2700E). All measurements were automated with LabView 2017.

## 3. Results

In this section, the response of two devices is analyzed. The difference between them lay in the specific number of printed CNTs layers. In the first case, the heater was made of only one layer, whereas the second device contained two printed sensing layers. Finally, the devices are compared with similar ones found in the literature.

### 3.1. Heater with One Printed CNT Layer

The considered structures can be modelled as a conducting film realized on top of a massive insulating substrate. As a consequence of this consideration, it is possible to model them through a 1D transient heat equation, where the Joule-effect generated heat is temperature dependent.

The heat source would be the resistive network of carbon nanotubes (whose interdependence with temperature can be easily estimated from the empirical curves), which is related to both the thickness of the layer and the current temperature, while the heat dispersion will be mostly due to convection.

A simple expression would be

$$\rho_{s}c_{s}\frac{\partial T}{\partial t}-\kappa \frac{\partial^{2}}{\partial x^{2}}=0$$

which in turn gives

$$-\kappa \frac{\partial }{\partial x}=h\left(T_{Top}-T_{0}\right)+q^{\prime \prime }$$

In this analytic description, $\rho_{s}$ is the density of the substrate, $c_{s}$ is its heat capacity, κ is its thermal conductivity, and h represents the heat exchange phenomena. Finally $q^{\prime \prime }=\frac{V^{2}}{R\left(T\right)\times S}$ is the heat source, where the voltage, the temperature dependent resistance, and the surface are identified with V, R(T), and S, respectively. These equations show how most of the time dependent temperature effect in the heaters lie in three classes of factors: the intrinsic thermal characteristics of the substrate, the exchange rate, and the resistance of the network. Given a set substrate material, the first set of parameters are fixed, leaving as degrees of freedom only h and the resistance. Concerning h, previous literature shows that it is not strongly influenced by the level of coverage (how many nanotubes are on the substrate) [26], hence—in the given material and substrate framework—the main variable that is driving the performance of the heater will its sheet resistance.

The resistance of low-density CNT networks is inversely proportional to temperature and broadly linear. The conduction mechanisms in such networks are complex but can be assimilated to temperature assisted carrier transport: as long as the temperature increases, the hopping efficiency will increase, until it reaches the theoretical maximum mobility. In a static environment, in which the number of carriers is not dramatically changing, the resistance should be stable at a constant point. However, what it is observed experimentally and well documented with computer-based simulations [16], is that the resistance decreases down to a transition point, in which the resistance change flattens and reverts. This transition point is mostly attributed to the thermally activated desorption of weakly bonded oxygen, with consequent de-doping of the network. Here, the lack of doping species shifts the average Fermi level in the CNT film, with consequent reduction of the available carriers and conductivity. Reaching this point results in the permanent increase of resistance of the CNT film, associated with an increased linearity and a shift of the turning point to higher temperature [16].

**Figure 2:**
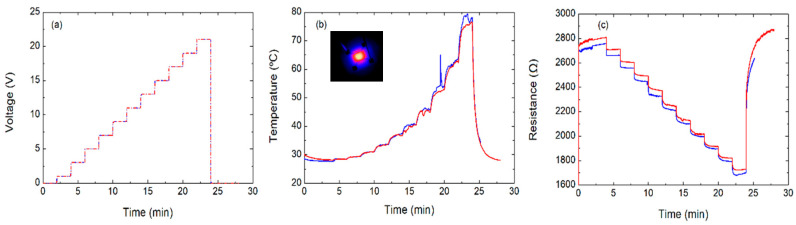
Characterization of the one-layer device applying a sweep voltage up to 21 V (two cycles): (**a**) voltage over time, (**b**) temperature over time (inset is a thermal image of the heater), and (**c**) resistance over time.

This characteristic can be also exploited to define a burn-in procedure, through which the temperature coefficient can be stabilized, and the film can be utilized in a more predictable manner. Firstly, this procedure was applied to the single-layer heater. For that, the device was subjected to a voltage sweep until the transition point was reached (19 V), which increased the initial resistance from 2100 Ω to 2700 Ω in ambient conditions. This burn-in procedure shifted the turning point to above 21 V, rendering the device predictably usable in the whole desired range. After that, two cycles of a voltage sweep from 0 V to 21 V in steps of 2 V every 2 min were applied to the sample, as depicted in Figure 2. Under these conditions, the main features extracted from these results are listed in Table 1.

The average maximum temperature was 78.10 ± 1.97 °C at the maximum applied voltage (21 V) with an average power consumption of 259 ± 6 mW, which means a power density of 81.13 ± 1.88 mW/mm^2^. Additionally, we also defined a figure of merit (FoM) to evaluate and compare more appropriately the performance of the haters in terms of power density required to achieve a certain temperature. Thus, the FoM was defined as indicated in Equation (1)

FoM = T_max_ · [A/P_Tmax_], (1)

with T_max_ being the maximum temperature, P_Tmax_ the power consumption at T_max_ and A the area of the heater. Therefore, the higher the FoM, the more efficient the heater is. In this case, we obtained a FoM of ~0.97 °C mm^2^ mW^−1^.

**Figure 3:**
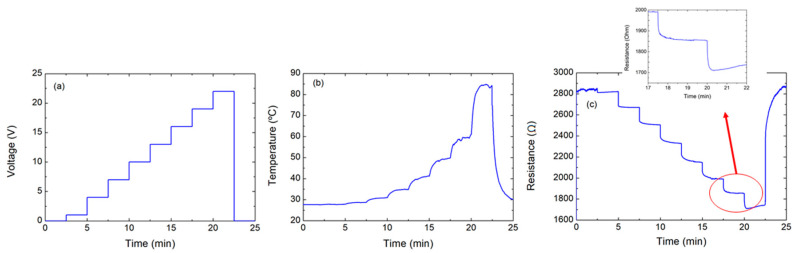
Characterization, applying voltage of 22 V: (**a**) voltage over time, (**b**) temperature over time, and (**c**) resistance over time.

In general, the deviation from average in the measurements was circa 3% for all the parameters. Moreover, Figure 3 shows how the transition point started to appear when the applied voltage was increased above 21 V. Consequently, after the recovery, the resistance increased up to 3240 Ω at ambient conditions (26 °C). After 3 days, the same experiments (22 V) were repeated. It resulted in virtually no transition point, whereby 82.12 °C was the maximum temperature achieved (see supplementary Figure S1). This clearly shown that the device could be burned-in to stabilize its behavior and increase its maximum temperature. However, the burn-in process also increased the basic resistance of the film: in order to achieve the same temperature, it will be necessary to apply increasing voltages after every burn-in process.

**Figure 4:**
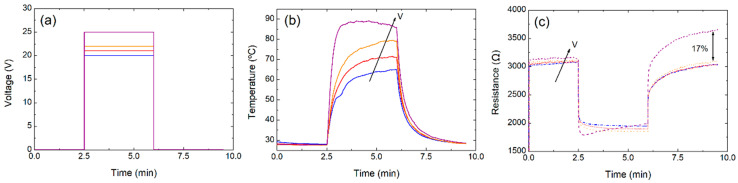
Characterization, applying different voltage levels (20 V, 21 V, 22 V, and 25 V): (**a**) voltage over time, (**b**) temperature over time, and (**c**) resistance over time (the arrow shows the increase in the voltage).

To understand the limitations of this procedure, the same structure was subjected to different voltage steps of 210 s (20 V, 21 V, 22 V, and 25 V). In these experiments, the transition point could be clearly distinguished at 25 V, as shown in Figure 4, while all other characteristic values are summarized in Table 2.

It can be noted that, once the transition point was achieved, the FoM suffered a notable decay, indicating that, although higher temperatures might be achieved, a higher voltage will be required. For that, when two new voltage steps of 22 V were applied again to the sample (see supplementary Figure S2), the results showed that the maximum temperature had been reduced down to 65 °C (in contrast to the 79.56 °C of the latter experiments), while the resistance at ambient conditions increased for about 20%.

**Figure 5:**
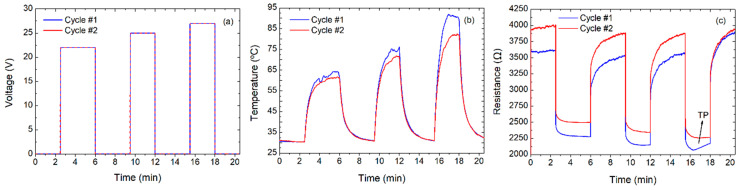
Three steps at different voltage levels. Characterization, applying three steps at different voltages: (**a**) voltage over time, (**b**) temperature over time, and (**c**) resistance over time. TP stands for Transition point; the arrow indicates when TP has shifted (and the temperature to achieve the TP has increased).

Finally, to prove that the transition point could be shifted again, two different cycles of voltage steps (22 V, 25 V, 27 V) were applied to the sample once more. Thus, as seen in Figure 5, while in the first cycle the transition point was clearly identified at 27 V, reaching a maximum temperature of about 75 °C at 25 V, the second cycle did not present transition point. In addition, as expected, in the second cycle the temperature at 25 V was reduced by 5 °C with respect to that of the first cycle.

This demonstrated that it is possible to progressively shift the turning point. However, this benefit on the maximum temperature is limited, and the trend reverts when the basic resistance exceeds a certain threshold. The most fitting burn-in strategy will be based on the particular needs of the applications, i.e., whether the generated temperature or the linearity shall be optimized.

### 3.2. Heater with Two Printed CNT Layers

**Figure 6:**
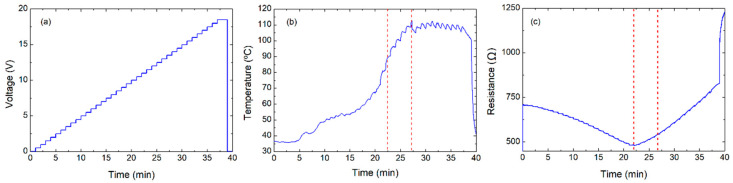
Heater with two printed CNT layers. Characterization, applying voltage up to 18.5 V: (**a**) voltage over time, (**b**) temperature over time, and (**c**) resistance over time.

The same procedure was followed for the characterization of the double-layer CNT heaters. In that case, the initial resistance of the two-layer heater was about 700 Ω, which corresponds to a value 3 times lower than the obtained for the single layer heater, as it was expected by increasing the number of CNT layers [27]. First, a voltage sweep from 0 V to 18.5 V with 0.5 V steps every 1 min was applied to the sample, as observed in Figure 6.

**Figure 7:**
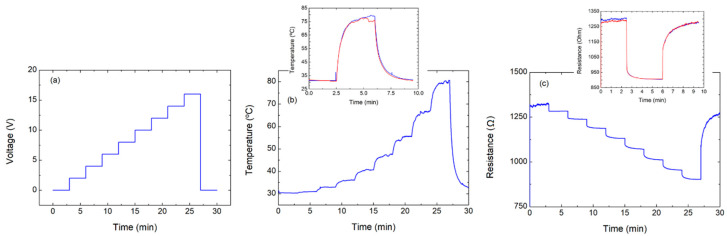
Sweeps up to 16 V after transition point. Characterization, applying voltage up to 16 V (after reaching the transition point): (**a**) voltage over time, (**b**) temperature over time, and (**c**) resistance over time. Insets correspond to the characterization, performed applying two steps at 16 V.

In that case, it can be noted how during the time interval 22.5–27.5 min, even though the resistance started to increase, the temperature remained increasing. Furthermore, the increasing of the resistance continued even after the temperature became stable, achieving a final value of about 1300 Ω after the recovery. The FoM calculated at 11 V, when the device reached a temperature of circa 100 °C is circa 1.37 °C mm^2^ mW^−1^, which is remarkably better than the case with a single CNT layer. However, voltage above 10 V clearly deteriorated the behaviour of the device, altering its base resistance. Thus, higher temperatures can be obtained, at the expenses of the heater’s efficiency. To confirm that the device would work reliably and repeatedly when the voltage is kept below the guard level, two new voltage sweeps up to 16 V were performed, whose results are depicted in Figure 7. As can be observed, no transition point was achieved in these tests, confirming that the burn-in procedure is successful also for the more densely populated networks. In any case, the maximum temperature was higher than that obtained for the single-layer devices, though lower voltages were applied. Furthermore, the transition temperature point is higher (about 37%) than in the first characterized device.

**Figure 8:**
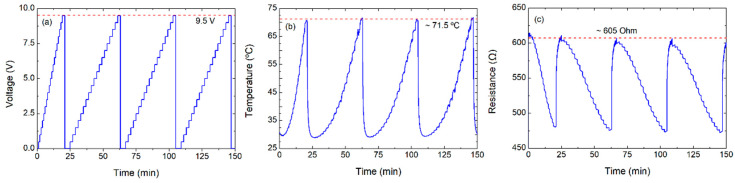
Characterization, applying voltage up to 9.5 V, for consecutive cycles: (**a**) voltage over time, (**b**) temperature over time, and (**c**) resistance over time.

To estimate the reproducibility of the heating mechanism and the FoM with heaters whose base resistance had not been deteriorated by excessive temperature, a new device with the same fabrication parameters was measured. As the transition point was expected to be above 10.5 V, a test varying the voltage was performed every minute from 0 to 10 V with 0.5 V steps (see supplementary Figure S3). The initial resistance of this device was 660 Ω (with a difference of 6% with respect to the first device with two printed layers). It can be observed that the transition point slightly appeared at 10 V, for a temperature of ~77 °C. Finally, the reproducibility of the heating characteristics of a two-layer device is demonstrated in Figure 8, which presents 4 cycles of a test varying the voltage every minute from 0 to 9.5 V in 0.5 V steps. The results show that a maximum stable temperature of about 71.5 °C can be achieved for a voltage of 9.5 V with a resistance of ~605 Ω (with an error below 3%). Therefore, the double-layer heaters presented a FoM of ~1.53 °C mm^2^ mW^−1^, which was higher than that obtained for their counterparts based on only one layer.

Finally, with respect to the heater stability, we observed virtually no variation if we kept the voltage below the transition temperature after 10 consecutive cycles with a variation lower than 0.5%. We also tested the heater performance measuring it daily during 10 consecutive days and the changes among measurements were also below 0.5%.

### 3.3. Comparison with Other Microheaters in the Literature

Finally, Table 3 presents a comparison between the heaters described in this work and other close related flexible microheaters. Different aspects should be considered regarding the performance of the flexible heaters, in order to establish an appropriate cost-efficiency analysis. Therefore, the heaters presented here were compared with respect to similar ones in terms of materials and fabrication processes, as well as operation point (area and both maximum temperature and input power). It can be noted that the printed CNTs-based heaters can reach a maximum temperature, which is comparable to the ones reported for other materials. Moreover, in the case of the double layer heaters, this maximum temperature is reached for lower values of applied voltage (up to 37% lower), which helps to work with low input powers. Higher performances could be achieved, if other materials such as Au are considered.

It can be noted that the inkjet-printed CNTs heaters proposed in this work present a figure of merit comparable to that obtained with other graphene-based heaters in the literature [28,29]. Moreover, the performance achieved with the ink-jet printed CNT heaters is of the same order of magnitude than the reported for similar CNT heaters in the literature. The clear advantage of the inkjet-printed heaters lay in their balance of cost effectiveness to performance. The inkjet-printed method is both simpler and cheaper than the photolithography or CVD methods; besides, it allows the fabrication of micro-patterned heaters, which is not approachable with other techniques in a cost-effective way.

Higher performances could be also achieved considering other materials or doping agents, such as Au [12]. However, in this case, material costs would pose a large obstacle for a commercial large-scale production of devices.

It should be noticed that although SWCNT can be still expensive, it is not necessary to achieve any specific chirality or aspect ratio, semiconducting/conducting mix, and purity for the heating application, which drastically reduces their cost.

## 4. Conclusions

In this paper, a simple method for the fabrication of fully printed flexible microheaters using CNTs as heating element is presented. The study has been carried out considering single CNT layer as well as a double-layer structure. The results have shown that a larger number of CNT layers implies a lower voltage to achieve the transition temperature. Furthermore, once the transition temperature is reached, the resistance after the recovery is increased. This, in turn, increases the voltage required to reach again the transition point and stabilizes the device. Through these observations, we are able to define a burn-in procedure, which helps the reliable employment of these printed heaters.

The proposed heaters exhibit temperatures above 70 °C at 9.5 V with a power consumption of 150 mW in an area of 3.2 mm^2^. This makes them stand out among other heaters reported in literature, for their efficient generation of high temperatures at low input powers. Besides that, the fabrication process is simple, cheaper, and can be carried out on diverse substrates. The employment of ink-jet printing makes the process compatible both with large structures and with millimeter-sized heaters inserted in complex printed structures. All these features, together with the roll-to-roll compatibility, make this technology applicable for large-scale production of devices for diverse applications, which require the use of heaters as a conformal patch.

## Figures and Tables

**Table 1:** Parameters extracted from Figure 2.

Parameters	Cycle #1	Cycle #2
Max temperature (°C)	79.49	76.70
Min temperature (°C)	27.64	28.03
Min resistance (Ω)	1679.6	1721.9
Max resistance (Ω)	2758.1	2876.9
Voltage (V)	21	21
Power density (mW mm^−2^)	82.05	80.04
FoM (°C mm^2^ mW^−1^)	0.97	0.96

**Table 2:** Parameters extracted from Figure 4.

Parameters	Cycle #1	Cycle #2	Cycle #3	Cycle #4
Max temperature (°C)	65.04	71.51	79.56	89.12
Min temperature (°C)	27.99	27.74	27.48	27.59
Min resistance (Ω)	1940.4	1895.5	1850.2	1789.5
Max resistance (Ω)	3088.9	3096.1	3127.8	3662.2
Voltage (V)	20	21	22	25
Power density (mW mm^−2^)	64.42	72.70	81.75	109.14
FoM (°C mm^2^ mW^−1^)	1	0.98	0.97	0.82

**Table 3:** Comparison among closely related microheaters

Reference	Materials	Fabrication Process	Area (mm^2^)	Max. Operating Temperature (°C)	Max. Power (mW)	FoM (°C mm^2^mW^−1^)
Wu et al. [28]	RGOH-Pt on Si/SiO_2_	Photolithography	1	140	192	0.73
Khan et al. [29]	Graphene on boron nitride (h-BN) sheets	CVD	0.096	200	39	0.5
Ilanchezhiyan et al. [20]	CNT on cotton fabrics	Dip Coating	-	52	320	7.69
Jung et al. [30]	MWCNT on glass	CVD	0.02	100	9600	0.2·10^−3^
Liu et al. [31]	CNTs on PET	CVD	4	100	120	3.33
Khan et al. [12]	AuNPs on polyimide	Aerosol printing	0.25	250	39	1.6
This work	SWCNT and AgNPs on PET	Inkjet printing	3.2	80 ± 10@22 V (one layer) 71.5 ± 0.5@9.5 V (two layers)	260 ± 50@22 V (one layer) 150 ± 10@9.5 V (two layers)	0.98 1.53

## Supplementary Materials

The following are available online at https://www.mdpi.com/2079-4991/10/9/1879/s1, Figure S1: Heater with one printed CNT layer. Characterization applying a step voltage of 22 V, comparison among the same test before and after reaching the transition point: (a) voltage over time, (b) temperature over time, (c) resistance over time and (d) power over time. Figure S2: Heater with one printed CNT layer. Characterization, applying two steps of 22 V (blue: first pulse, red: second pulse). (a) Voltage over time, (b) temperature over time, (c) resistance over time, and (d) power over time. Figure S3: Characterization, applying voltage up to 10 V: (a) voltage over time, (b) temperature over time, (c) resistance over time.
