# Supplementary material for: Printed and Flexible Microheaters Based on Carbon Nanotubes
Source: Nanomaterials (Basel). 2020 Sep 19;10(9):1879. doi: 10.3390/nano10091879 (PMC7558360; doi:10.3390/nano10091879)
Supplement: Supplementary file 1 [file nanomaterials-10-01879-s001.pdf]

nanomaterials

Supplementary Materials

Printed and Flexible Microheaters Based on Carbon Nanotubes

Aniello Falco [1], Francisco J. Romero [2], Florin C. Loghin [3], Alina Lyuleeva [4], Markus Becherer [3], Paolo Lugli [1], Diego P. Morales [2], Noel Rodriguez [2], Jose F. Salmerón [3], and Almudena Rivadeneyra [2,*]

[1] Faculty of Science and Technology, Free University of Bolzano, 39100 Bolzano-Bozen, Italy; aniello.falco@unibz.it (A.F.); paolo.lugli@unibz.it (P.L.)
[2] Department of Electronics and Computer Technology, University of Granada, 18071 Granada, Spain; franromero@ugr.es (F.J.R.); diegopm@ugr.es (D.P.M.); noel@ugr.es (N.R.)
[3] Institute for Nanoelectronics, Technical University of Munich, 80333 Munich, Germany; florin.loghin@tum.de (F.C.L.); markus.becherer@tum.de (M.B.); jf.salmeron@tum.de (J.F.S.)
[4] Department of Electronical and Computer Engineering, Rice University, Houston, 77005 TX, USA; alina.lyuleeva@rice.edu
* Correspondence: arivadeneyra@ugr.es; Tel.: +34-958-248-996

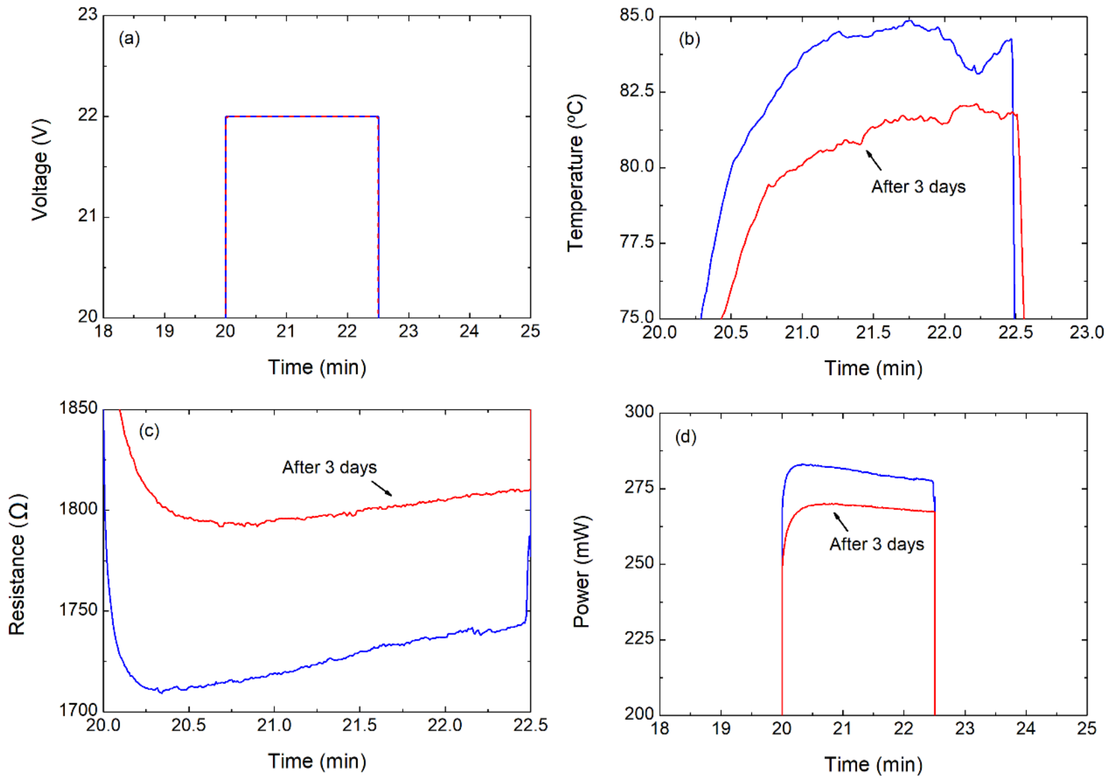

Figure S1. Heater with one printed CNT layer. Characterization applying a step voltage of 22 V, comparison among the same test before and after reaching the transition point: **(a)** voltage over time, **(b)** temperature over time, **(c)** resistance over time and **(d)** power over time.

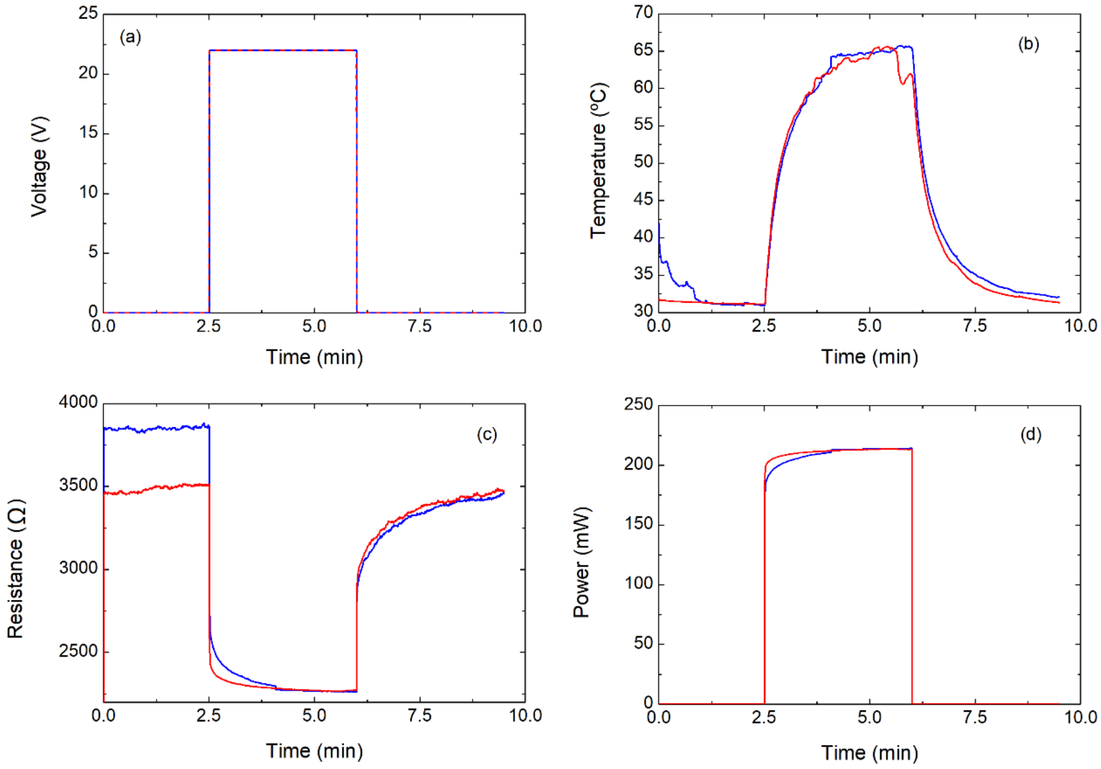

Figure S2. Heater with one printed CNT layer. Characterization, applying two steps of 22 V (blue: first pulse, red: second pulse). **(a)** Voltage over time, **(b)** temperature over time, **(c)** resistance over time and **(d)** power over time.

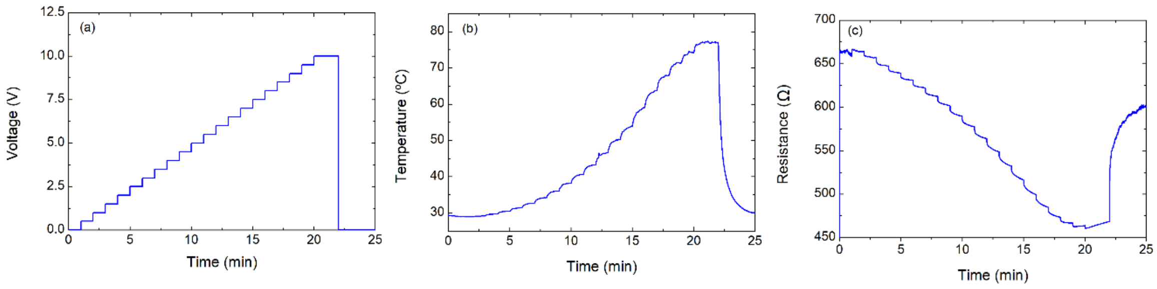

Figure S3. Characterization, applying voltage up to 10 V: **(a)** voltage over time, **(b)** temperature over time, **(c)** resistance over time.
